# Prediction of the First Variceal Haemorrhage

**DOI:** 10.1155/1997/37863

**Published:** 1997

**Authors:** K-J Paquet

**Affiliations:** Lessingstrasse 8 Bad Kissingen D-97688 Germany

## Abstract

We followed 87 cirrhotic patients with esophageal varices and without previous hemorrhage for a mean period of 24 mo to prospectively evaluate the occurance of variceal bleeding within (early) or after (late) 6 mo from entry and the contribution of portal Doppler ultrasound parameters to the prediction of early and late hemorrhage. Clinical, biochemical, endoscopic and portal Doppler ultrasound parameters were recorded at entry. Variceal bleeding occurred in 22 patients (25.3%). Nine (40.9%) bled within the first 6 mo. Cox regression analysis identified variceal size, cherry-red spots, serum bilirubin and congestion index of the portal vein (the ratio of portal vein [cross-sectional area] and portal blood flow velocity) as the only independent predictors of first variceal hemorrhage. Discriminant analysis was used to find the prognostic index cut off points to identify patients who bled within 6 mo (prognostic group 1) or after 6 mo (prognostic group 2) or remained free of bleeding (prognostic group 3). The cumulative proportion of patients correctly classified was 73% in prognostic group 1, 47% in prognostic group 2 and more than 80% in prognostic group 3. The addition of Doppler ultrasound flowmetry to clinical, biochemical and endoscopic parameter only improved the classification of patients with early bleeding.


Although not widely availble when this trial was initiated in 1978, endoscopic treatment (sclerosis and banding) has become the mainstay in most centers for management of the acute variceal bleeder with bleeding control rates in excess of 70% in nearly all series[4,5,6]. Additionally, other effective non-operative therapies such as transjugular intrahepatic portosystemic shunt (TIPS)[7] and intravenous octreotide[4,8] have been introduced since the completion of this trial. Since a TIPS accomplishes portal decompression in a manner similar to a surgical shunt, it would be expected to relieve variceal bleeding as well, with potentially less morbidity, especially in high risk patients. Patients treated with TIPS often improve their liver function, but those who deteriorate due to inadequate functional hepatic reserve can be considered for liver transplantation. Unlike patients with surgical shunts, TIPS does not increase, and in fact may decrease the risk of liver transplantation.

Since the results of this trial, which enrolled patients from 1978 to 1983 but which was not published until 1994, may have limited relevance to the management of acute variceal bleeding in 1996, the results of an ongoing trial of EPCS versus endoscopic treatment by Orloff's group are eagerly awaited. Will this provide a definitive answer? Probably not, because TIPS is now available for high-risk patients who fail emergency endoscopic treatment. In fact, it is unlikely that any single therapy will ever be uniformly applicable to this heterogeneous group of patients. Now that several effective modalities are available, a thoughtful, individualized approach is essential to obtain optimal results.

### *References*

[1] Cello, J.P., Grendell, J.H., Crass, R.A., Weber, T.E., and Trunkey, D.D. (1987). Endoscopic sclerotherapy versus portacaval shunt in patients with severe cirrhosis and acute variceal hemorrhage. *N. Engl. J. Med.*, **316**, 11–15.

[2] Paquet, K.J. and Feussner, H. (1985). Endoscopic sclerosis and esophageal balloon tamponade in acute hemorrhage from esophagogastric varices: a prospective controlled randomized trial. *Hepatology*, **5**, 580–583

[3] Holman, J.M. and Rikkers, L.F. (1980). Success of medical and surgical management of acute variceal hemorrhage. *Am. J. Surg.*, **140**, 816–820.

[4] D'Amico, G., Pagliaro, L. and Bosch, J. (1995). The treatment of portal hypertension: meta-analytic review. *Hepatology*, **22**, 332–354.

[5] Westaby, D. and Williams, R. (1990). Status of sclerotherapy for variceal hemorrhage in 1990. *Am. J. Surg.*, **160**, 32–36.

[6] Stiegmann, G.V., Go, J.S., Michaletz-Onody, P.A., Korula, J., Lieberman, D. and Saeed, Z.A., *et al.* (1992). Endoscopic sclerotherapy as compared with endoscopic ligation for bleeding esophageal varices. *N. Engl. J. Med.*, **326**, 1527–1532.

[7] Ring, E.J., Lake, J.R. Roberts, J.P., Gordon, R.L., LaBerge, J.M. and Read, A.E., *et al.* (1992). Using transjugular intrahepatic portosytemic shunt to control variceal bleeding before liver transplantation. *Ann Intern. Med.*, **116**, 304–309.

[8] Besson, I., Ingrand, P., Person, B., Boutroux, D. and Bernard, P., *et al.* (1995). Sclerotherapy with or without octreotide for acute variceal bleeding. *N. Engl. J. Med.*, **333**, 555–60.

Stuart J. Knechtle, M.D. and
Layton F. Rikkers, M.D.
Department of Surgery
University of Wisconsin Hospital and Clinic
H4/710 Clinical Science Center
600 Highland Avenue
Madison, WI 53792
Tel: (608) 263-1377
Fax: (608) 265-5963

### Prediction of the First Variceal Haemorrhage

### ABSTRACT

*Siringo, S., Bolondi, L., Gaiani, S., Sofia, S., Zironi, G., Rigamonti, A., Di Febo, G., Miglioli, M., Cavalli, G. and Barbara, L. (1994). Timing of the first variceal hemorrhage in cirrhotic patients: Prospective evalua-*

*tion of doppler flowmetry, endoscopy and clinical parameters. Hepatology;* **20**: *66–73.*

**We followed 87 cirrhotic patients with esophageal varices and without previous hemorrhage for a mean period of 24 mo to prospectively evaluate the occurance of variceal bleeding within (early) or after (late) 6 mo from entry and the contribution of portal Doppler ultrasound parameters to the prediction of early and late hemorrhage. Clinical, biochemical, endoscopic and portal Doppler ultrasound parameters were recorded at entry. Variceal bleeding occurred in 22 patients (25.3%). Nine (40.9%) bled within the first 6 mo. Cox regression analysis identified variceal size, cherry-red spots, serum bilirubin and congestion index of the portal vein (the ratio of portal vein [cross-sectional area] and portal blood flow velocity) as the only independent predictors of first variceal hemorrhage. Discriminant analysis was used to find the prognostic index cut off points to identify patients who bled within 6 mo (prognostic group 1) or after 6 mo (prognostic group 2) or remained free of bleeding (prognostic group 3). The cumulative proportion of patients correctly classified was 73% in prognostic group 1, 47% in prognostic group 2 and more than 80% in prognostic group 3. The addition of Doppler ultrasound flowmetry to clinical, biochemical and endoscopic parameter only improved the classification of patients with early bleeding. (Hepatology 1994; 20: 66–73.)**

*Keywords*: Variceal haemorrhage cirrhosis portal hypertension, oesophageal varices

### PAPER DISCUSSION

Approximately, 25 to 30% of cirrhotic patients with esophageal varices and without previous variceal hemorrhage will bleed from ruptured varices and 70% of them will do so within the first two years of follow-up [1]. However, studies evaluating the risk factors for the first variceal bleeding have not assessed the timing of the variceal bleeding during this period [2–4]. The assessment of these patients suggest [5] that the risk of bleeding within the first two years is not constant; it tends to decrease after an inital period of six months. Therefore, the identification of risk factors for early and late bleeding is important because it could allow randomization of patients into different treatment groups in prophylactic trial for the prevention of a first variceal hemorrhage, i.e. by betablockers or endoscopic sclerotherapy.

Risk factors for a high bleeding risk have been described by our group [6], by the Japanese Research Society for Portal Hypertenion[7] and by the North Italian Endoscopy Club[2]. However, in these description the role of portal hemodynamics in the prediction of variceal bleeding are not included. The New Haven Group [8] has described the prevalence of a certain level of portal pressure for the risk of variceal bleeding. Therefore, this additional risk factor was included in a prospective randomized trial of prophylactic endoscopic sclerotherapy of our group and seems to be very useful [9].

However, this selection criterion is invasive and thus has limits. Doppler ultrasound is a new tool for evaluating portal hypertension, providing a non invasive assessment of blood flow in most splanchnic vessels. The current use of doppler ultrasound has focused on studies of pathophysiology of portal hypertention and on assessment of effects of vasoactive drugs on splanchnic blood flow. Early studies of portal hemodynamics in cirrhotic patients may have a potential prognostic value in assessment of the risk of variceal bleeding [12].

Therefore, the group of Siringo prospectively evaluated the (a) occurrence of the first variceal bleeding, defined hemorrhage occurring within six months of entry into control studies, and late first variceal bleeding (i.e. bleeding occuring after six months of follow-up), and (b) the role of portal doppler ultrasound parameters, in addition to clinical and endoscopic criteria, in predicting the early and late occurrence of hemorrhage, as well as the possibility of patients remaining free of bleeding.

Therefore, during the period of 38 months, 95 consecutive cirrhotic patients with esophageal varices and without previous variceal hemorrhage were subjected to doppler ultrasound evaluation. The doppler ultrasound was technical feasible in 87 patients (91,6%), who were subseqently followed up for a mean period of 24,0 (±14,5) months. 71 (81.6%) of the 87 were inpatients when they entered the study. At entry, the patients underwent upper gastrointestinal endoscopy, and varices were classified according to the Japanese Research Society of portal hypertension endoscopic rule [7]. Clinical and biochemical data were recorded in each patient, and the severity of the liver disease was assessed using the Cambell numeral modification of Child's grading[13].

The following portal hemodynamic parameters were evaluated in the 87 patients by means of real-time ultrasound equipment with a 3,5-MHz-convex transducer with a pulse doppler device working at 3,5 and 2,5 MHz frequencies:

a) Diameter of the portal vein (PV) (mm) was calculated as the anteroposterior diameter during suspended respiration at the largest point.

b) Blood flow velocity of the portal vein (PV) (cm/min.) was calculated with the equipment from the doppler spectrum described by Gill[14]. The mean velocity ($V_{mean}$) of portal flow was measured. The sample volume, as large as at least half of the vessels caliber was positioned 1cm distal to the crossing point of the hepatic artery with the portal trunk in an oblique, subcostal scan.

c) Portal blood flow volume (PFV) (mm/min.) was calculated by the formula FVF=$V_{mean} \bullet \pi \bullet r^2$ where $r$ represents the diameter of the vessel.

d) Congestion index of the portal vein (CI): ratio of cross-sectional area and the blood flow velocity in the portal vein. The CI was calculated according to a modified Moriyasus's formula (15) using the $V_{mean}$ instead of the maximal flow velocity.

$$CI = \frac{(r)^2 \bullet \pi/10}{V_{mean}}$$

where $r$ is the vessels radius. For example, a portal vein diameter of 16mm and a portal flow velocity of 12 cm/min. yields a CI of 1.67.

In additon, the group of Siringo searched for spontaneous portal-systemic shunts and portal vein thrombosis in all patients. These two variables were referred to as morphological ultrasonographic findings.

The diagnosis of variceal hemorrhage was made when after an episode of hematemesis, melena or both emergency endoscopy (within 72 hours of the clinical manifestation of bleeding or index bleed), showed (a) active bleeding, a clot or a "white nipple" on a varix; (b) no lesions potentially responsible for bleeding when varices lacked the previously described signs; or (c) potential sources of hemorrhage other than varices but without signs of active or recent bleeding. Patients who did not undergo emergency endoscopy were considered to have bled from an unknown source. In this case the varices were considered the source of bleeding.

Thereafter, an univariate and multivariate analysis and a development of a prognostic model was performed including the mentioned risk factors. In this discriminant analysis the prognostic index, cut off points were found to identify patients who bled within 6 months (prognostic group I), after 6 six months (prognostic group II) or remained free of bleeding (prognostic group III). The cumulative proportion of patients correctly classified was 73% in the prognostic group I, 47% in the prognostic group II, and more than 80% in the prognostic group III. The addition of doppler ultrasound flowmetry to clinical, biochemical and endoscopic parameters only improves the classification of patients with early bleeding.

Thus, the cumulative rate of bleeding within 6 months in the group with early bleeding, as predicted by clinical and endoscopic criteria, was only 54%, quite poor and similar to 58%, as predicted by the North Italian Endoscopy Index of more than 40%[2]. The cumulative rate of actual bleeding predicted in this group was significantly improved (19% more) by the Congestion Index. However, the identification of patients with late occurrence of bleeding (after 6 months from entry) was poor in both models, with or without the inclusion of the Congestion Index (45 vs. 50%).

In conclusion, the study of Siringo *et al.*[5] shows, that a subgroup of cirrhotic patients is at a high risk of bleeding within 6 months of entry into the study. This subgroup of patients is best identified by a prognostic model based on clinical, endoscopic and doppler parameters. Patients who bled after 6 months from entry are poorly identifiable when only the status at entry is analyzed. Thus in this group the Congestion Index only poorly improved the bleeding risk criteria introduced by the North Italian Endoscopy Club (2). However, the criteria described by our group[9] are more reliable.

### *References*

[1] Burroughs, A.K., D'Heygere, F. and McIntyre, N. (1986). Pitfalls in studies of prophylactic therapy for variceal bleeding in cirrhotis. *Hepatology* **6**, 1407–1413

[2] North Italian Endoscopic Club for the Study and Treatment of Esophageal Varices:(1988). Prediction of the first hemorrhage in patient with cirrhosis of the liver and esophageal varices. *N. Engl. J. Med.* **319**, 983–989.

[3] Piai, G., Minieri, M., Catalano, M., D'Angelo, V., Sauro, A., Sabbatini, F. and Mazzacca, G. (1991). A prospective validation of two indexes of first variceal bleeding in liver cirrhosis (Abstract). *Gastroenterology* **100**, A784.

[4] Kleber, G., Sauerbruch, T., Ansari, H. and Paumgartner, G. (1991). Prediction of variceal hemorrhage in cirrhosis: a prospective follow-up study. *Gastroenterology* **100**, 1332–1337.

[5] Siringo, S., Bolondi, L., Gaiani, S., Sofia, S., Zironi, G., Rigamonti, A., DiFebo, G., Miglioli, M., Cavalli, G. and Barbaram, L. (1994). Timing of the first variceal hemorrhage in cirrhotic patients: prospective evaluation of doppler flowmetry, endoscopy and clinical parameters. *Hepatology* **2**, 66–73.

[6] Paquet, K.-J. (1982). Prophylactic endoscopic sclerosing treatment of the esophageal wall in varices – a prospective controlled randomized trial. *Endoscopy* **14**, 4–5.

[7] Japanese Research Society for Portal Hypertension. (1980). The general rules for recording endoscopic findings on esophageal varices. *Jpn. J. Surg.* **10**, 84–87

[8] Garcia-Tsao, G., Groszmann, R., Fisher, R., Conn, H., Atterbury, C. and Glickman, M. (1985). Portal pressure, presence of gastroesophageal varices and variceal bleeding. *Hepatology* **5**, 419–424.

[9] Paquet, K.-J., Kalk, J.-F., Klein, C.-P. and Gad, H.A. (1994). Prophylactic sclerotherapy for esophageal varices in high-risk cirrhotic patients selected by endoscopic and hemodynamic criteria: a randomized, single-center controlled trial. *Endoscopy* **26**, 734–740.

[10] Bolondi, L., Gaiani, S. and Barbara, L. (1991). Doppler flowmetry: clinical applications in portal hypertensive patients. In: Okuda K., Benhamou J-P, eds. Portal hypertension. Springer-Verlag, Tokyo, 161–182.

[11] Siringo, S., Di Febo, G., Bolondi , L., Gaiani , S., Vacirca, M., Miglioli, M. and Barbara, L. (1990). Predicting variceal bleeding in cirrhotics using endoscopy, hemodynamic evaluation by doppler ultrasound (DUS) and clinical variables (Abstract) *Gut* **31**. A1209.

[12] Gaiani, S., Bolondi, L., LiBassi, S., Zironi, G., Siringo, S. and Barbara L. (1991). Prevalence of spontaneous hepatofugal portal flow in liver cirrhosis: Clinical and endoscopic correlation in 228 patients. *Gastroenterology* **100**, 160–167.

[13] Cambell, D., Parker, D. and Anagnostopulos, C. (1973). Survival prediction in portacaval shunts: a computerized statistical analysis. *Am J. Surg* **126**, 748–751

[14] Gill, R. (1979). Pulsed doppler with B-mode imaging for quantitive blood flow measurement. *Ultrasound Med. Biol.* **5**, 223–235.

[15] Moriyasu, F., Nishida, O., Ban, N., Nakamura, T., Sakai, M., Miyake, T. and Uchino, H. (1986). "Congestion Index" of the portel vein. A J R (*Am J Roentgenol*) **146**, 735–739.

K-J Paquet, MD
Professor of Surgery
Lessingstrasse 8
D-97688 Bad Kissingen
Germany

